# Hybrid Solid Polymer Electrolytes Based on Epoxy Resins, Ionic Liquid, and Ceramic Nanoparticles for Structural Applications

**DOI:** 10.3390/polym16142048

**Published:** 2024-07-18

**Authors:** Bianca K. Muñoz, Jorge Lozano, María Sánchez, Alejandro Ureña

**Affiliations:** 1 Material Science and Engineering Area, ESCET, Universidad Rey Juan Carlos, C/Tulipán s/n, 28933 Móstoles, Spain; j.lozanoma@alumnos.urjc.es (J.L.); maria.sanchez@urjc.es (M.S.); alejandro.urena@urjc.es (A.U.); 2 Instituto de Tecnologías Para la Sostenibilidad, Universidad Rey Juan Carlos, C/Tulipán s/n, 28933 Móstoles, Spain

**Keywords:** solid polymer electrolyte, composite polymer electrolyte, structural supercapacitor, energy storage, structural devices

## Abstract

Solid polymer electrolytes (SPE) and composite polymer electrolytes (CPE) serve as crucial components in all-solid-state energy storage devices. Structural batteries and supercapacitors present a promising alternative for electric vehicles, integrating structural functionality with energy storage capability. However, despite their potential, these applications are hampered by various challenges, particularly in the realm of developing new solid polymer electrolytes that require more investigation. In this study, novel solid polymer electrolytes and composite polymer electrolytes were synthesized using epoxy resin blends, ionic liquid, lithium salt, and alumina nanoparticles and subsequently characterized. Among the formulations tested, the optimal system, designated as L70P30ILE40Li1MAl2 and containing 40 wt.% of ionic liquid and 5.7 wt.% of lithium salt, exhibited exceptional mechanical properties. It displayed a remarkable storage modulus of 1.2 GPa and reached ionic conductivities of 0.085 mS/cm at 60 °C. Furthermore, a proof-of-concept supercapacitor was fabricated, demonstrating the practical application of the developed electrolyte system.

## 1. Introduction

Multifunctional structural supercapacitors and batteries are considered an outstanding approach for energy storage applications in electrical vehicles (EVs) [1,2]. It is well-known that reducing weight in a vehicle can help reduce fuel consumption. This statement has also been estimated in terms of energy consumption (around 7%) when weight is reduced by 10% [3,4]. Based on this fact, electrical vehicles have different options for reducing weight, the most intuitive and well-established consist of using lightweight materials (fiber composites or aluminum matrix composites) [5]. Another potential way includes structural materials as energy storage devices, the most attractive route but still far from practical implementation and commercialization [4,5]. The synergic combination of lightweight-resistant materials as carbon fiber-reinforced polymers (CFRP), with their ability for energy storage (supercapacitors, batteries, and fuel cells), and detaching the inert battery mass (needed but useless) appears to be an ideal scenario for achieving a practical fuel independency [6]. Concerning the components of a multifunctional device, recent studies have been addressed regarding the modification of carbon fiber as electrodes, the development of solid polymer electrolytes (SPE), the optimization of glass or Kevlar fiber separators, the coupling of current collectors, etc. 

The SPE are key components of the structural supercapacitors and batteries, and they are at their early stage of development since their ionic conductivities are still not comparable to liquid electrolytes. In this context, different approaches have been carried out so far with various degrees of success, revealing some technical issues, as the stability window and ionic conductivity, that still need to be solved in this technology before being considered as a competitive option. Hybrid electrolytes (HE) or composite polymer electrolytes (CPE) are electrolytes with more than two components that can be an alternative to traditional electrolytes to simultaneously improve ionic conductivity and mechanical properties. They have been classified as active and passive electrolytes, depending on the nature of their reinforcements and whether they participate or not in the conduction mechanism. Electrolytes with ceramic reinforcements that do not provide ionic movement by themselves, such as SiO_2_ or TiO_2_, are considered passive. Passive HEs have recently attracted the attention of researchers because they exhibit higher mechanical resistance and thermal stability than active HEs, and being less expensive and more accessible [7,8,9,10,11]. For instance, alumina has been reported to modify the ionic conductivity in polymer electrolytes by preventing the formation of crystals and improving mechanical properties due to their rigid nature [12,13,14,15]. Some examples of its use have been reported in poly(vinyl alcohol) (PVA) matrices, where it has been observed that using nano-alumina in 6 wt.% showed a remarkable increase in conductivity due to the increase of amorphous areas in the PVA. Above 6 wt.%, aggregation of alumina NPs that creates unsuitable paths for ion transfer has been detected [12]. Also, many studies have used alumina as reinforcement in a poly(ethylene oxide) (PEO) matrix, including lithium salt [16,17].

Epoxy resins are appropriate matrices for structural applications [18]. Those derived from Polyethylene Glycol Diglycidyl Ether (PEGDGE) or diglycidyl ether of bisphenol A (DGEBA) have been investigated as SPEs [19,20,21,22,23]. Kwon et al. reported the introduction of alumina nanowires (2–6 nm) in a epoxy resin/Li salt system. They have observed that increasing the amount of alumina used to increase the conductivity (0.29 mS·cm^−1^ at 25 °C), but above certain content can result in reducing the ionic dissociation due the NPs aggregation. Thus, a very high alumina content acts in detriment to the *T_g_* of the resulting polymer [14]. Another successful example has been reported by Choi and coworkers, where SPEs based on DGEBA, succinonitrile and lithium salt afford ionic conductivities from 1 × 10^−3^ to 0.1 mS/cm, reaching a high Young’s modulus of 1 GPa in some cases [24].

Recently, in our group, we have developed HEs based on a mixture of epoxy resins with PEGDGE, and a commercial epoxy resin (Araldite LY556) based on DGEBA, using a commercial hardener Araldite XB3473 and 4,4′-diaminodiphenyl sulfone. The additives used were the ionic liquid 1-Ethyl-3-methylimidazolium bis(trifluoromethylsulfonyl)imide (EMIMTFSI) and titania nanoparticles (21 nm). These electrolytes exhibited good mechanical properties (*T_g_* > 70 °C and storage modulus at 30 °C > 1 GPa), as well as promising ionic conductivities [20]. Using the same epoxy resin system, a systematic study was carried out including alumina (particle size < 13 nm, 2–8 wt.% of nanoparticles content) [25]. In this study the best system found, containing 2 wt. % of alumina, was able to afford outstanding thermomechanical properties (*T_g_* = 83 °C and E′ at 30 °C = 1.2 GPa) and modest ionic conductivities at room temperature (σ_1_ = 1.6 × 10^−3^ mS/cm). This family of electrolytes was found to be very appropriate for structural applications, as it has been demonstrated in the fabrication of a structural supercapacitor using graphene-modified woven carbon fiber and woven lass fiber as separator, which exhibit a Young modulus of 24.0 ± 1.6 GPa and a tensile strength of 294 ± 9 MPa [26]. From a mechanical point of view, the CFRP obtained with this electrolyte formulation achieves a promising behavior for energy storage devices since, in some practical examples previously reported for batteries, the Young modulus and tensile strength are in the range of 1.8–25 GPa and 90–300 MPa, respectively [27]. 

Despite the good results obtained using the SPEs mentioned above [25], these formulations cannot be used for structural Li-ion batteries as they do not contain lithium ions.

Based on this limitation, herein we report a new family of SPEs and CPEs for structural supercapacitors and lithium-ion batteries. The new formulation of SPEs and CPEs, are also based on epoxy resin blends, ionic liquid, lithium salt, and alumina nanoparticles. This new electrolyte family represents a promising alternative in the development of more efficient and safer structural energy storage devices.

## 2. Materials and Methods

### 2.1. Materials

Solvents and reagents were purchased and used without further purifications. Epoxy resins Araldite LY556 based on DGEBA and its hardener XB3473, were purchased in Huntsman. The epoxy resin has an epoxy equivalent mass range of 183.48–188.67 epoxy equiv^−1^. The amines equivalent mass range of the crossing agent was 82.6–89.3 g amine equiv^−1^. The ratio Epoxy resin/hardener used was 100:23 wt.%, according to the supplier recommendations, and this system was identified as L. The reagents Poly(ethylenegycol) Diglycidyl Ether (PEGDGE, Mn 500), 4,4′-diaminodiphenyl sulfone (DDS), the lithium salt bis(trifluoromethylsulfonyl)imide lithium (LiTFSI, Li), propylene carbonate (PC), alumina nanoparticles (Al, 13 nm), copper chloride, (L)-ascorbic acid (L-AA), and PVP K90 were purchased from Sigma-Aldrich (St. Louis, MO, USA). Ionic liquid 1-ethyl-3-methyl-imidazolium bis(trifluoromethylsulfonyl) imide (ILE, 99%) was purchased from IOLITEC -Ionic Liquids Technologies GmbH (Heilbronn, Germany). The resin system PEGDGE/DDS used an epoxy/amine ratio of 100:35 wt.%, and it was identified as P. Woven carbon fabric HexForce^®^ 48,193 plain 12 K, manufactured by Hexcel (Stamford, CT, USA), was used in this work. The glass fiber separator used (E-Fiberglass Woven Roving) was purchased from Castro Composites^®^ (Pontevedra, Spain).

The nomenclature used for the reagents and epoxy resins are denoted in bold capital letters, using the acronyms shown above.

### 2.2. Methods

All the samples were characterized by dynamic mechanical thermal analysis (DMTA) Q800 V7.1 (TA Instruments, New Castle, DE, USA), cyclic voltammetry (CV, AUTOLAB PGSTAT302N, Herisau, Switzerland), linear sweep voltammetry (LSV, AUTOLAB PGSTAT302N, Herisau, Switzerland), electrochemical impedance spectroscopy (EIS, AUTOLAB PGSTAT302N, Herisau, Switzerland) and field-emission gun scanning electron microscopy (FEGSEM) Nova NanoSEM 230 apparatus from Philips (Amsterdam, The Netherlands).

#### 2.2.1. Dynamic Mechanical Thermal Analysis (DMTA)

The thermo-mechanical properties were studied following ASTM 5418 [27] in a single cantilever mode over a temperature range from 25–275 °C, using a ramp of 2 °C/min and a frequency of 1 Hz. The thermal scanning was performed from −50 to 200 °C, to determine the glass transition temperature (*T_g_*), which was measured as the maximum of the loss tangent curve (tan δ). In addition, the average modulus at room temperature allows evaluating the stiffness of the samples. 

#### 2.2.2. Cyclic Voltammetry (CV) and Linear Sweep Voltammetry (LSV)

The CV and LSV analyses were performed to determine the stability window and electrochemical behavior of the electrolytes. The measurements were performed in a potentiostat AUTOLAB PGSTAT302N with a software Nova 2.1. The CV parameters were from −1 to 1 V at 10 mVs^−1^ and for LSV the potential varied from 0 to 4 at the same scan rate. 

#### 2.2.3. Electrochemical Impedance Spectroscopy (EIS)

The samples for EIS were dried overnight in an oven at 80 °C after being painted with silver ink to favor the electrode–electrolyte contact. The EIS analysis was performed in a potentiostat AUTOLAB PGSTAT302N. The samples were sandwiched between two symmetric clean and polished stainless-steel electrodes and introduced in a homemade press hold sampler [25], to improve the electrolyte–electrode contact. The impedance of the samples was measured at room temperature using a frequency range between 1.0 MHz and 0.1 Hz and an amplitude of 30 mV. The Nyquist plots (imaginary contribution of impedance Z″ vs. real contribution Z′) showed a semicircle at high frequencies and an additional contribution at lower frequencies corresponding to the R_0_ (CPE_1_ R_1_) (CPE_2_ R_2_) equivalent circuit. The ionic conductivities were obtained from R_1_ for the solid polymer electrolytes following Equation (1). The composite polymer electrolytes (those containing alumina nanoparticles) showed two conduction paths due to the reinforcement and two ionic conductivities were obtained from R_0_ and R_1_, respectively.

(1) $$\sigma =\frac{d(cm)}{R(\Omega )\times A({cm}^{2})}$$

#### 2.2.4. Chronoamperometry for Lithium Transference Number Calculations (*t*_Li_^+^)

The samples were sanded to obtain thin layers. The layers were dried in an oven and placed under vacuum overnight before preparing the coin cell battery CR2032 in Li/CPE/Li configuration. The coin cells were assembled in the glove box. The lithium transference number was measured following Bruce–Vincent–Evans equation at room temperature (2), where ΔV is 10 mV, I_0_ and I_SS_ the initial and steady state currents, and R_0_ and R_SS_ are the resistances obtained from EIS analysis before and after polarization. The coin cells were tested by dc polarization, applying 10 mV during 7200 s. EIS analysis to determine the ionic conductivity were acquired before and after the chronoamperometry.

(2) $${t_{Li}}^{+}\frac{I_{SS}(∆V-R_{0}I_{0})}{I_{0}(∆V-R_{SS}I_{SS})}$$

#### 2.2.5. Field-Emission Gun Scanning Electron Microscopy (FEGSEM)

The electrolyte-free samples were analyzed morphologically using FEGSEM Nova NanoSEM 230 working at 5 kV and at 5 mm of distance. The samples were treated previously with ethanol to remove the ionic liquid following the procedure described in the literature [22]. This procedure included changing the solvent twice a day, for one week and drying the samples in a vacuum oven at 70 °C overnight. Cryofractured samples were analyzed after coating with gold in a gold sputtering (2 nm Au).

### 2.3. Solid Polymer Electrolyte Preparation

The solid polymer electrolytes were prepared following two different procedures described below, depending on whether the samples contain nanoparticles or not. The weight of reagents was calculated for 30 g of the total sample according to the wt.% content listed in Table 1. Once the samples were cured, they were prepared to adapt the form for each technique samplers. 

Procedure A (for samples without nanoparticles): In a flat bottom jar the lithium salt was dissolved in the ionic liquid. Then, both resins L and P were weighed in the same jar and the mixture was degassed under vacuum at 80°C for 15 min. Next, the hardeners XB3473 and DDS were added, and the mixture was stirred under vacuum for 10 more minutes. The mixture was placed on a metallic mold and left to cure at 140°C for 8 h, as previously described.

Procedure B (for samples containing Al_2_O_3_ nanoparticles): In a flat bottom jar the lithium salt was dissolved in the ionic liquid. Alumina was added to the previous solution and the nanoparticles were dispersed by ultrasonication in a Hielscher ultrasonic processor UP400 St at 0.5 pulse cycles and 50% amplitude for 1.5 h. Then, both resins L and P were weighted in the same jar and the mixture was degassed under vacuum at 80 °C for 15 min. Next, the hardeners DDS and XB3473 were added, and the mixture was stirred under vacuum for 10 more minutes. The mixture was placed on a metallic mold and left to cure at 140 °C for 8 h, as previously described.

### 2.4. Supercapacitor Fabrication

The electrodes of CuO NPs on woven carbon fiber were fabricated following the procedure previously reported in the literature [28]. For this, 1 wt.% of PVP K90 was added to a solution containing CuCl_2_/NaCl/L-Ascorbic acid (molar ratio of 1:3.5:15) in deionized water (DIW, 375 mL). The pH was adjusted to 3.9 using NaOH, and this solution was used as a growth solution for the hydrothermal modification of CuO on WCF. The reaction was left in the autoclave at 80 °C for 2 h. The fibers obtained were washed with DIW and dried overnight in the oven at 60 °C to use it as electrodes. The structural supercapacitor was fabricated by vacuum-assisted resin infusion molding (VARIM) as reported [24] and using the CPE L70P30(ILE40)Li(Al2). The composite was cured in an oven at 140 °C for 8 h. The supercapacitors were composed of two layers of WCF electrodes with a two-layer GF separator between them. Copper sheets were also attached to each CFRP electrode by using silver ink to ensure a good electrical contact. The EIS test was carried out on the composites in a frequency range of 10 × 10^6^–0.1 Hz. The tests were also performed in an AUTO- LAB PGSTAT302N module with Nova 2.1 software.

## 3. Results and Discussion

### 3.1. Thermomechanical and Morphologycal Characterization of the Electrolytes

In this study, samples of electrolytes were prepared keeping in mind the ability to enhance the electrochemical and mechanical properties simultaneously. We studied the effect of increasing the amount of ionic liquid (40, 45, and 50 wt. % of ILE) and the structural resin content (60, 65, and 70 wt. % of L resin). The effect of the addition of PC (5 wt.%) and ceramic nanoparticles (2 wt.% of alumina, Al2) was also studied. Table 2 shows the mean values of the storage modulus (E′), loss modulus (E″), and glass transition temperature (*T_g_*), as well as their standard deviations obtained through the DMTA analysis.

Concerning the amount of ionic liquid included (40, 45, or 50 wt.%), it was possible to observe a higher storage modulus (E′) at lower ionic liquid content for all the formulations. The highest values were obtained for those samples containing 40% of ILE (from 539 to 1032 MPa at 30 °C, entries 1–3). Surprisingly, the *T_g_* values do not vary in this regard, being around 70 °C for the samples L70P30 containing 40, 45, and 50 wt.% of ILE (entries 1, 4, and 7), meaning that these values are more susceptible to the resin’s ratio (L/P) than to the ionic liquid content. The same behavior was observed for the *T_g_* values of samples L65P35 and L60P40 (entries 2, 5, and 8 and 3, 6, and 9, respectively). Regarding the amount of the more structural resin (L, from 60 to 70 wt.%), when increases up to 70% the *T_g_* and the storage modulus (E′) were also found to increase (entries 1–9), as was expected and observed in our previous study [25]. From a mechanical perspective, the samples containing 70% L resin appear to be the best suited for structural applications, since as soon as the L content is below 70 wt.%, the storage modulus drops irremediably.

The incorporation of PC was then studied in two formulations, L70P30(ILE40)Li and L65P35(ILE40)Li (entries 10 and 11), and it was observed that there was a more remarkable drop in the storage modulus for the resin L70P30(ILE40) (from 1032 to 603 MPa) than for the resin with a lesser L content (from 659 to 504 MPa).

This additive was only added to samples containing 40% ILE to avoid an extreme loss of storage modulus. The *T_g_* were lower, decreasing 7 °C in both samples. Alumina nanoparticles were then added to samples with formula L70P30(ILEX)Li (entries 12–14), to improve and recover mechanical strength. The sample with 40 wt.% of ILE (entry 12), exhibits the best improvement with a 1.23 GPa of storage modulus. For the other samples (45 and 50 wt.% ILE), this effect was not so remarkable, as the values were practically the same as without nanoparticles. The *T_g_* values with the addition of nanoparticles slightly increased in the three L/P resin’s ratio studied.

**Figure 1:**
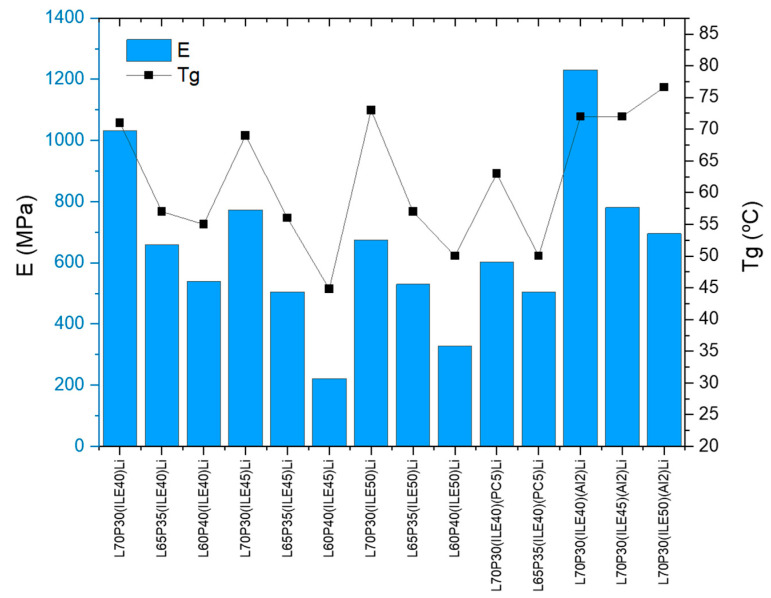
Storage modulus (E′, blue bars) and glass transition temperature (*T_g_*, black squares) of the samples.

Figure 1 shows the results of storage modulus E′ and *T_g_* for all of the samples studied. It can be observed that when alumina (2 wt.%) is included in the electrolytes, L70P30, for 40, 45, and 50% ILE, slightly increases the *T_g_* of the samples regarding the initial values. The best sample performance was found for the sample containing 40% ILE and alumina, L70P30(ILE40)Li(Al2) (entry 12).

### 3.2. Electrochemical Characterization of Electrolytes

All the samples were analyzed by EIS to calculate the ionic conductivity. The values are listed in Table 3 (the Nyquist plots equivalent circuit fittings are shown in Supplementary Materials, Figures S1–S5).

Before discussing ionic conductivity, it is necessary to understand the ionic movement in hybrid electrolytes with non-active fillers [29]. The ionic movements in a HE can occur via a hopping mechanism through the polymer chain, where the Li^+^ and other cations hop though the oxygen atoms in ethylene glycol polymer chains (-O-CH_2_-CH_2_-O-); this is usually known as bulk polymer conductivity (σ_1_). The other way the ions can move is by percolation through the interfacial layer, that can be defined as that region were the conducting dispersing medium (Li in ILE) is immobilized or confined on the surface of the nanofiller [30]. In this article, this interfacial ionic conductivity will be referred to as interfacial conductivity (σ_0_), and it will be calculated only for those electrolyte formulations including alumina.

The ionic conductivities of all the polymer electrolytes prepared in this work are between 2.6 × $10^{-7}$ and 5.7 × $10^{-6}$ S/cm. In general, as we decrease the L content in the resin blends, the ionic conductivities increase due to the more elastomeric nature of resin P, which favors the ionic movements through the polymer chains. This behavior has been observed in other blends where there are two or more resins with a different nature [20].

The addition of ionic liquid also affects the ionic conductivities; for 40 wt.%, the samples exhibit the lowest ionic conductivity values (~$\times 10^{-7}$ S·cm^−1^, entries 1–3). These values can be improved by at least one order of magnitude when a plasticizer such as propylene carbonate (PC) is added, rising to 1.2 × $10^{-6}$ S·cm^−1^ (entry 11). On the contrary, the samples containing 50 wt.% of ionic liquid did not show the best ionic conductivity values, being between 7.2 × $10^{-7}$ and 6.1 × $10^{-6}$ S·cm^−1^ (entries 7–9). For these samples, an exudate of ionic liquid appears after a few days, which means that so much ionic liquid could reduce the stability of the electrolyte in operation.

According to the electrochemical stability range, values obtained from the LSV tests (Table 3) were found to increase with greater ionic liquid and lithium salt content. Thus, the samples with 40 wt.% of ionic liquid (7.6 wt.% Li) showed the lowest stability, followed by those with 45% and 50 wt. %, containing (8.5 and 9.4 wt.% of Li, respectively). Similar behavior has been reported for polymer electrolytes containing ionic liquid and lithium salts [31,32]. This improvement can be attributed to the synergic effect of lithium salt in ionic liquids. 

**Figure 2:**
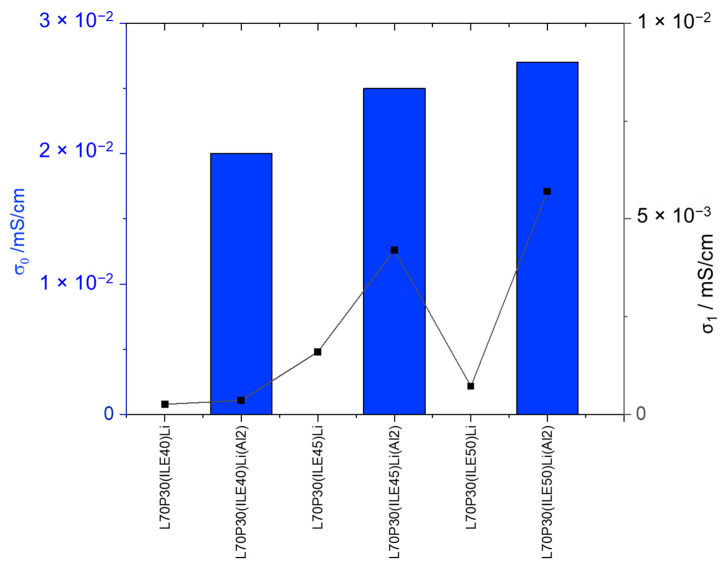
Ionic conductivities of the electrolytes with and without alumina nanoparticles.

The introduction of alumina nanoparticles improves the ion mobility through the bulk, in all cases, and the interfacial ionic conductivity obtained shows much higher values (Table 3, entries 12–14, and Figure 2). Moreover, the electrochemical window is greater, meaning the addition of alumina nanoparticles also enhances the electrochemical stability of the electrolytes. 

In general terms, the specific capacitance values (shown in Table 3) increase within each percentage of ionic liquid (40, 45, and 50 wt.%), as the percentage in P resin is greater. This result is justified since the mobility of the ions through the polymer chains is greater as the number of groups of ethylene oxide units (EO), present in the P resin, increases. This favors ionic transport, improving the electrochemical properties of the electrolyte.

**Figure 3:**
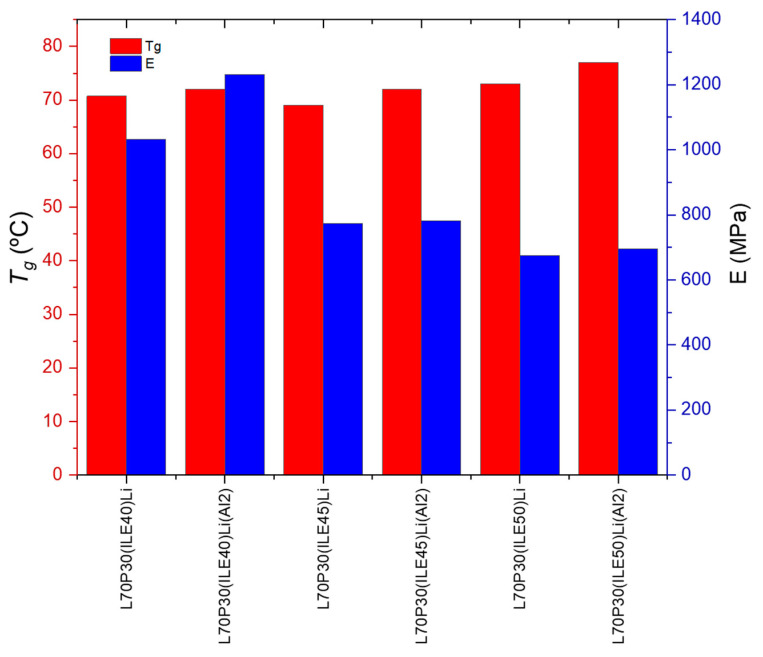
Thermomechanical parameters of the electrolytes with and without alumina nanoparticles.

To discuss the best systems found with the addition of alumina nanoparticles, regarding not only the electrochemical behavior but the mechanical properties, a representation of the storage modulus and the *T_g_* for samples with and without the reinforcement is shown in Figure 3. The only sample that reaches E′ > 1 GPa is that containing 40 wt. % of ionic liquid and 7.6 wt.% of lithium content; then, at higher ionic liquid content, the storage modulus drops dramatically.

Concerning the *T_g_*, the addition of alumina nanoparticles increases the values, which is more remarkable for the samples with 40% ionic liquid. From a structural point of view, the best equilibrium was obtained for the sample L70P30(ILE40)LiAl2, where the modulus and *T_g_* are very high with a modest ionic conductivity. 

**Figure 4:**
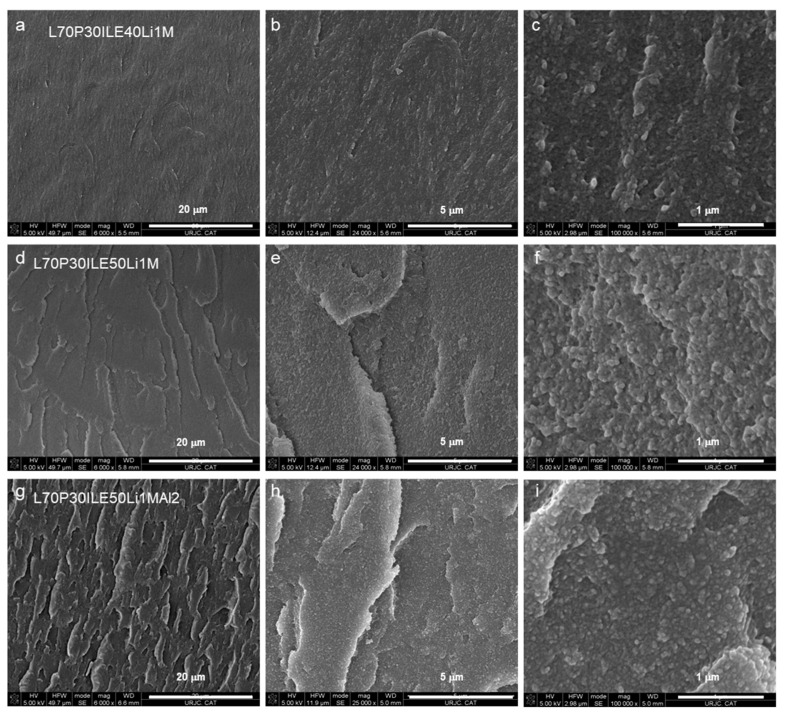
FEGSEM images at different magnifications of the epoxy resins (**a**–**c**) L70P30(ILE40)Li, (**d**–**f**) L70P30(ILE50)Li, and (**g**–**i**) L70P30(ILE50)LiAl2.

Some samples were analyzed using FEGSEM to evaluate the differences associated with the ionic liquid content and the addition of nanoparticles. Figure 4a–c, Figure 4d–f, and Figure 4g–i show images at different magnifications of cryofractured samples of L70P30(ILE40)Li, L70P30(ILE50)Li and L70P30(ILE50)LiAl2, respectively. The microstructure of the crosslinked resins with different ionic liquid contents (40 and 50 wt.%) is entirely amorphous and does not exhibit either a biphasic or porous structure, even at the highest ionic liquid content. We have observed that the sample with a lower ionic liquid content (Figure 4a–c) has a smoother and less rough surface at the micrometric level compared to samples containing 50% ionic liquid (Figure 4d–f). The ionic liquid facilitates plastification mechanisms, activating mechanisms of crack deviation and fibrillation. The incorporation of nanoparticles reduces the size of the granular structures observed on the fracture surfaces (Figure 4c,f,i).

When alumina nanoparticles were introduced in the formulation (Figure 4g–i), an even rougher surface was obtained. For this sample, at higher magnification (Figure 4i) the nanoparticles seem to be very well dispersed and there is no evidence of big agglomeration or nanoparticle clusters on the surface. More stress concentration points were created, and the cracks interacted with the nanoparticles interface, increasing the number of cavities on the fractured surface. These stress points or defects are used to improve the ionic conductivity of the solid as it has been previously observed (Figure 2). For similar epoxy resin systems, a better nanoparticles dispersion improves the interfacial interaction between the reinforcement and the matrix [14], which explains the enhancement of the *T_g_* in all the cases studied [25].

In a previous study performed using the same epoxy resin system and ionic liquid, the sample L65P35(ILE30)Al2 exhibited the best balance between electrochemical and mechanical properties [25]. To compare the effect of the addition of lithium salt and alumina simultaneously in this formulation, another set of samples was prepared (Table 4). As it has been previously observed, the addition of lithium salt can have a positive effect on the stability of the electrolyte [20], and it was also observed in our system where the storage modulus had increased when the lithium salt was added (Table 1, entries 2 and 5 vs. Table 4, entries 2 and 3). However, in those cases the *T_g_* was slightly lower when lithium was included. The addition of alumina increases the *T_g_* of the electrolytes, which is more remarkable for those samples with a lower ionic liquid content (30 wt.%, Table 4 entries 1 and 4). The best ionic conductivity values were obtained for those samples containing alumina (Table 4, entries 4–6) compared to the plain samples without either lithium salt or nanoparticles (Table 4, entries 1–3). The simultaneous addition of lithium salt and alumina improves the storage modulus and the ionic conductivity in comparison to the samples modified only with lithium salt (Table 4, entries 7 and 8), values only surpassed by the sample containing alumina (Table 4, entries 4 and 8). A detrimental effect on the ionic conductivity with the addition of lithium salt has been previously observed [18,27], in some cases where the lithium concentration in an imidazole-based ionic liquid solution is low (<0.3 M) or high (>1.0 M) [33]. Some authors associate this effect with an increase in viscosity and a reduction in the mobility of ions in the electrolyte [19,33,34,35]. Choi and coworkers have found that the lithium salt (LiTFSI) concentration within the electrolyte mixture plays a crucial role in the formation of a bicontinuous nanoscale ion channel confined in the epoxy matrix, changing the ionic and epoxy domains [24].

**Figure 5:**
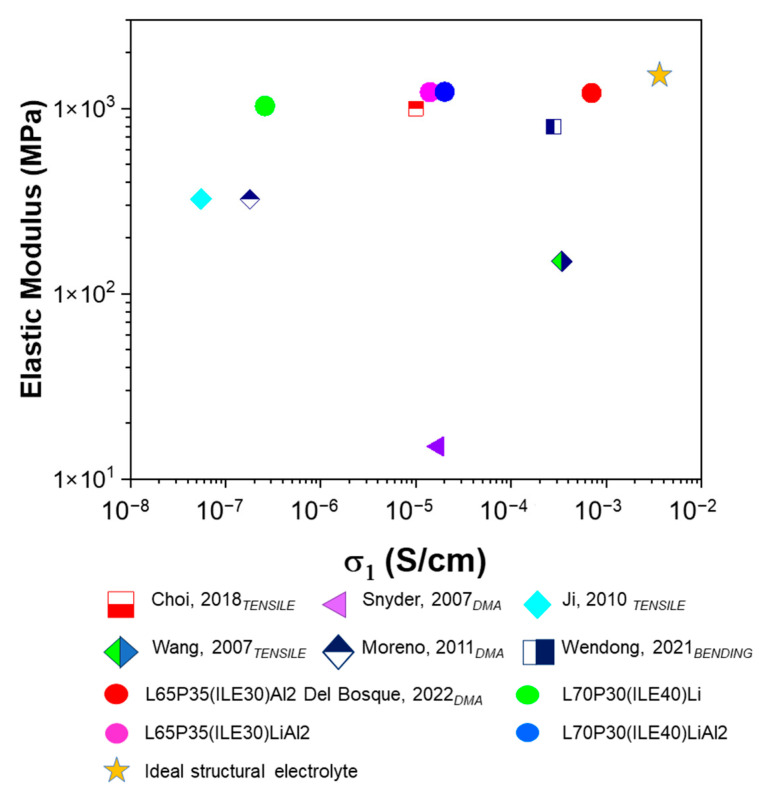
Mechanical and electrochemical comparison of the electrolytes. The subscript shows the mechanical test performed to establish the elastic modulus. Choi, 2018 [24]; Snyder, 2007 [24], Ji, 2010 [36], Wang, 2007 [37], Moreno, 2011 [38], Wendong, 2021 [21]; Del Bosque, 2022 [25].

A comparison of the elastic modulus and ionic conductivities of the SPEs/CPEs developed in this work with some of the best representative systems found in the literature [21,24,36,37,38,39] is depicted in Figure 5. 

The yellow star indicates ideal values of ionic conductivity for a neat ionic liquid and the Young modulus for a structural matrix based on epoxy resins. The colored circles are the electrolytes developed in this work. The green circle corresponds to our system L70P30(ILE40)Li without alumina, which is very robust. In the samples containing alumina (pink and blue circles), the ionic conductivity exhibits a very competitive value. The best CPE previously developed using the same resin blend but without lithium is L65P35(ILE30)Al2 (red circle) [25]. This electrolyte was very close to the ideal performance, but it does not contain lithium ions, so it cannot be used for structural batteries. Thus, the idea to reproduce an electrolyte for LIBs with this outstanding electrochemical and mechanical behavior was not possible only with the incorporation of lithium salt. In general, these electrolytes constitute a promising alternative considering the ionic conductivities obtained using less than 10% lithium as well as the competitive values in storage modulus.

The effect of temperature on the ionic conductivities of the best systems found in this study, L65P35(ILE30)Al2 and L70P30(ILE40)Al2, was checked. The samples were heated in an oven and connected to the potentiostat, and after reaching the appropriate temperature, the samples were led to equilibrate for a certain time (30 min). EIS analyses were measured to calculate the ionic conductivities through the impedances.

**Figure 6:**
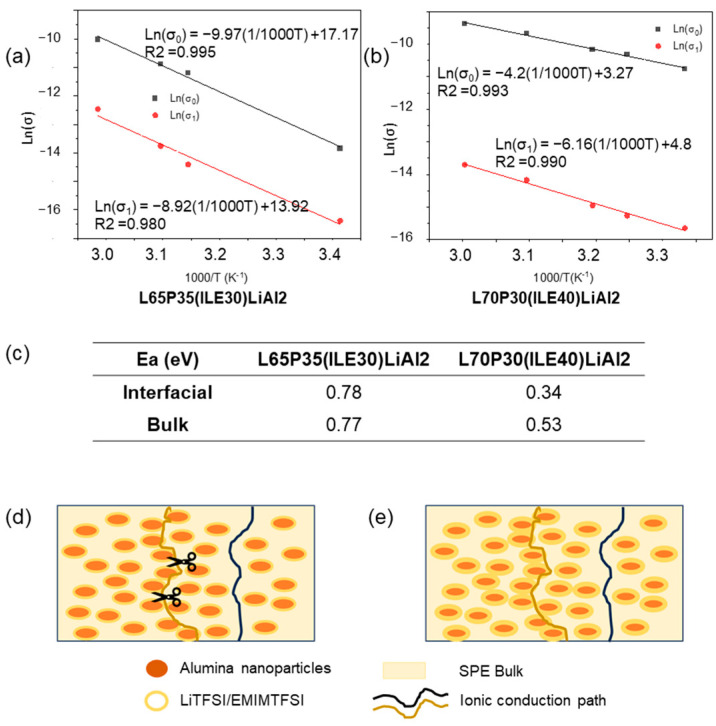
(**a**) Arrhenius type ionic conduction mechanism for L65P35(ILE)30LiAl2 and (**b**) for L70P30(ILE)40LiAl2 ionic conductivities in the electrolytes, including (**c**) activation energy in eV for the two ionic conductivities, (**d**,**e**) conduction paths in the CPEs through the bulk (black line), and percolation through the interfacial region (ochre line). Scissors represent the lost in the ionic conduction path due to the poor interfacial region in lower ionic liquid content electrolytes.

The Ln of ionic conductivity values (those corresponding to the bulk and interfacial regions) were plotted against the inverse of temperature (1000/T(K^−1^). The plots are shown in Figure 6a,b. Both composite electrolytes were fitted to the Arrhenius model, and the activation energies were calculated and shown in Figure 6c. 

For the sample L65P35(ILE30)LiAl2, the two activation energy values are higher than those obtained for the sample L70P30(ILE40)LiAl2; for the two ion-conduction paths, the values obtained for the sample L65P35(ILE30)LiAl2 were the same. Regarding the system L70P30(ILE40)LiAl2, two different values for the activation energy were obtained, and the activated energy associated with the interfacial layer was smaller, which means that the ionic movements associated with the Li/ions in the bulk are favored. These results can be explained if it is considered that the lithium salt is dissolved in an ionic liquid and this mixture is able to form a thicker layer on the nanoparticles surface (Figure 6d,e). In Figure 6e, the blue line path is the lithium ionic conduction through the bulk, as in typical SPEs. The ochre line path corresponds to the lithium ionic conduction way by a percolation through the interfacial layer, as it has been previously proposed for hybrid electrolytes [29,30]. For L70P30(ILE40)LiAl2, as the content of the ionic liquid is higher, the ions can move more easily compared to the ions hopping through the bulk.

The lithium transference number of electrolyte L70P30(ILE40)LiAl2 was calculated, and it was found to be *t*_Li_^+^ = 0.13. This number is usually related to the availability of free lithium in the mixture [40,41]. For systems based on PEO and LiTFSI, when the ratio of EO:Li is over three the *t*_Li_+ decreased dramatically [42]. In our case, the EO units (from PEGDGE) gave an EO:Li ratio of 14:1, meaning that the whole network is more hindered and there are countless interactions between lithium and its environment (anions, alumina NPs, etc.) that can explain the low value obtained. Nevertheless, the ionic conductivity that can be obtained for this system at 60 °C is 0.085 mS/cm, closer to the optimum value expected for a solid electrolyte.

### 3.3. Supercapacitors Performance of Composite Polymer Electrolytes

To test the applicability of the developed CPEs, we fabricated a structural supercapacitor using carbon fiber electrodes modified with CuO nanoparticles, following a procedure previously reported in our group [28]. In this procedure, woven carbon fiber (10 × 10 cm, approximately) was placed in a reactor containing a copper precursor (CuCl_2_) and some additives (NaCl, PVP K90, and L-AA) to favor the nanoparticles growing process. The hydrothermal process was left to react at 80 °C for 2 h. 

**Figure 7:**
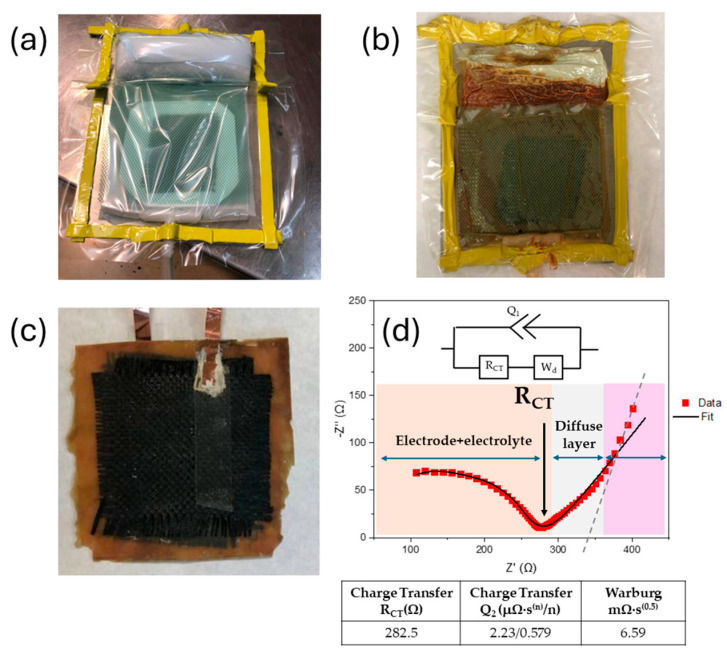
Sequence for the fabrication of the structural supercapacitor. (**a**) VARIM set-up before resin infusion. (**b**) VARIM after cured in an oven 8 h at 140 °C. (**c**) Structural supercapacitor demolded. (**d**) EIS Nyquist plot and equivalent circuit showed inset.

After this time, the woven carbon fiber was removed, washed with distilled water, and dried in an oven at 60 °C overnight. These modified carbon fibers have been successfully used as electrodes for flexible supercapacitor using a PVA/H_3_PO_4_ matrix as gel electrolyte [28]. The symmetric supercapacitor was assembled by piling up layers of modified carbon fiber, glass fiber, glass fiber, and modified carbon fiber, in that order, and introduced in a vacuum bag for the VARIM process (Figure 7a). The electrolyte L70P30(ILE40)LiAl2, chosen as the best option for the supercapacitor fabrication, was prepared following procedure B, described in Section 2.3. The quantities of resins weighted were recalculated for a total amount of 60 g.

Figure 7b,c shows the vacuum bag after the curing process, and the demolded structural composite after copper current collectors were glued using silver ink. The device was characterized by EIS, which shows an incomplete, depressed semicircle at high frequencies followed by an irregular spike at low frequencies. The data were fitted to a simple modified Randles circuit (typical for most EDLC). The equivalent circuit fitted is composed by a constant phase element (CPE) and in parallel a resistance and a Warburg impedance connected in series (Figure 7d, Table S1). Even when the fitted is very good, the interpretation of these values can be quite confusing, and they sometimes do not represent a real physical meaning. Mei and coworkers have established a physical interpretation of Nyquist plots without relating them to an electric circuit [43]. According to their findings, they could identify regions of electrode–electrolyte resistances (orange region in Figure 7d), the equilibrium differential capacitance (pink region in Figure 7d), and a diffusion layer resistance (gray region in Figure 7d), where the slope can be used to indicate if the charging process can be controlled by EDL formation or by ion diffusion in the electrolyte. Our device has a slope of 0.64 (R^2^ = 0.997), suggesting that the process is limited by ion diffusion, as it was expected, considering the nature of the CPE used. In the equilibrium differential capacitance region (pink in Figure 6d), a pure capacitive behavior exhibits a completely vertical line. Relating these results to the fitted equivalent circuit, the constant phase element (Q), as a non-ideal capacitor, denoted a mostly capacitive behavior (n~0.6) but was still far from an ideal capacitor. This interpretation agrees with the system studied, since the lithium transference number was found to be low enough to exhibit outstanding performance.

**Figure 8:**
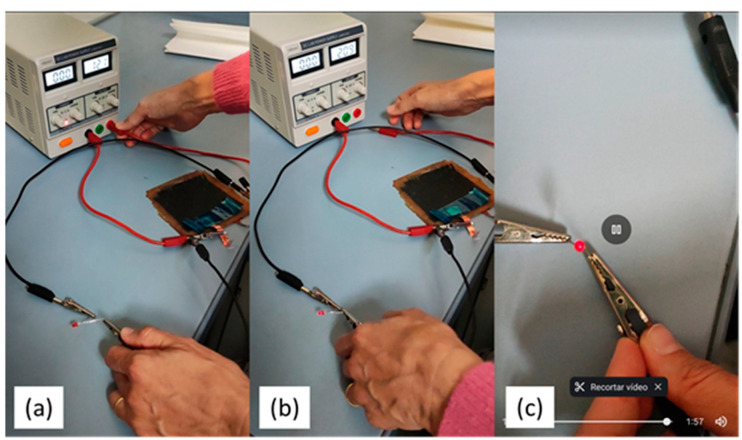
Sequence of the proof of concept by switching on a red LED (**a**) charging the device. (**b**) Switching on the red LED. (**c**) Red LED on after almost 2 min.

Finally, to carry out the proof-of-concept, the device, CuO-WCF/WGF/CuO-WCF embedded in the L70P30(ILE40)LiAl2 matrix, was connected to a power source for charging (Figure 8a) at 12 V for 2 h. Then, the device was disconnected from the power source (Figure 8b) and successfully powered a red LED light, but only for a couple of minutes (Figure 8c).

Attempts to manufacture a structural supercapacitor and battery completely inside the glove box in a pouch cell are currently in progress.

## 4. Conclusions

New SPEs and CPEs based on epoxy resin blends using 1-ethyl-3-methyl-imidazolium bis(trifluoromethylsulfonyl)imide ionic liquid, lithium bis(trifluoromethylsulfonyl)imide salt, and alumina nanoparticles (13 nm) were prepared, and the effect of resin composition (60–70 wt.% of structural based on DGEBA) and the ionic liquid content (from 30 to 50 wt.%) were studied. The resin blends containing 40 wt.% of ionic liquid were found to be optimal without observing any trace of exudate. The mechanical properties were improved by increasing the amount of DGEBA-based epoxy resin (**L**) in their final composition. Regarding the electrochemical properties, the addition of alumina nanoparticles was found to increase the ionic conductivities, and this effect was attributed to the interfacial alumina–electrolyte interaction in the polymer matrix. We have reached good thermal and mechanical properties for systems L70P30, higher than other similar systems previously reported (*T_g_* > 70 °C, E’ > 1.2 GPa). The new formulation found fulfills not only the requirements of a SPE, but it is also a cheaper alternative, considering it is fabricated using commercially available epoxy resins, and the ionic conductivity reported here is higher than other electrolytes with less than 10 wt.% lithium salt.

## Figures and Tables

**Table 1:** Composition of hybrid solid polymer electrolytes.

	wt.%					
Sample	L	P	ILE	Li (1M)	PC	Al
L65P35(ILE30)Li	41.80	22.50	30	5.7	-	-
L70P30(ILE40)Li	36.70	15.70	40	7.6	-	-
L65P35(ILE40)Li	34.20	18.20	40	7.6	-	-
L60P40(ILE40)Li	31.47	20.93	40	7.6	-	-
L70P30(ILE45)Li	33.55	12.95	45	8.5	-	-
L65P35(ILE45)Li	30.23	16.27	45	8.5	-	-
L60P40(ILE45)Li	27.90	18.60	45	8.5	-	-
L70P30(ILE50)Li	28.41	12.19	50	9.4	-	-
L65P35(ILE50)Li	26.40	14.20	50	9.4	-	-
L60P40(ILE50)Li	24.37	16.23	50	9.4	-	-
L70P30(ILE40)(PC5)Li	33.20	14.20	40	7.6	5	-
L65P35(ILE40)(PC5)Li	30.80	16.60	40	7.6	5	-
L65P35(ILE30)Li(Al2)	40.50	21.8	30	5.7	-	2
L70P30(ILE40)Li(Al2)	35.30	15.10	40	7.6	-	2
L70P30(ILE45)Li(Al2)	31.15	13.35	45	8.5	-	2
L70P30(ILE50)Li(Al2)	27.01	11.59	50	9.4	-	2

**Table 2:** Thermomechanical properties obtained by DMTA analysis.

Entry	Sample	*T_g_* (°C)	E′ (MPa) (T = 30 °C)	E″ (MPa) (T = 30 °C)
1	L70P30(ILE40)Li	70.7 ± 0.9	1032 ± 41	94 ± 6
2	L65P35(ILE40)Li	57 ± 2	659 ± 27	108 ± 8
3	L60P40(ILE40)Li	55 ± 2	539 ± 87	104 ± 17
4	L70P30(ILE45)Li	69 ± 2	773 ± 127	71 ± 2
5	L65P35(ILE45)Li	56 ± 3	506 ± 93	82 ± 4
6	L60P40(ILE45)Li	44.8 ± 0.5	222 ± 19	72 ± 3
7	L70P30(ILE50)Li	73 ± 1	675 ± 81	61.8 ± 0.9
8	L65P35(ILE50)Li	57 ± 4	530 ± 145	73 ± 14
9	L60P40(ILE50)Li	50 ± 2	328 ± 79	74 ± 12
10	L70P30(ILE40)(PC5)Li	63 ± 2	603 ± 122	76 ± 7
11	L65P35(ILE40)(PC5)Li	50 ± 1	504 ± 26	101 ± 5
12	L70P30(ILE40)Li(Al2)	72 ± 2	1231 ± 84	112 ± 5
13	L70P30(ILE45)Li(Al2)	72 ± 2	781 ± 77	74 ± 3
14	L70P30(ILE50)Li(Al2)	76.6 ± 0.8	696 ± 18	69 ± 1

**Table 3:** Electrochemical data for the solid electrolytes.

Entry	Sample	σ_0_ (S·cm^−1^)	σ_1_ (S·cm^−1^)	C_sp_ (μF/cm^2^)	Stability Range (V)
1	L70P30(ILE40)Li		$2.6\times 10^{-7}$	0.30	0.3
2	L65P35(ILE40)Li		$2.9\times 10^{-7}$	1.74	0.3
3	L60P40(ILE40)Li		$3.5\times 10^{-7}$	3.76	0.8
4	L70P30(ILE45)Li		$1.6\times 10^{-6}$	21.22	1.2
5	L65P35(ILE45)Li		$1.5\times 10^{-6}$	44.03	1.0
6	L60P40(ILE45)Li		$2.6\times 10^{-6}$	109.39	1.7
7	L70P30(ILE50)Li		$7.2\times 10^{-7}$	29.64	2.2
8	L65P35(ILE50)Li		$8.8\times 10^{-7}$	18.44	1.1
9	L60P40(ILE50)Li		$6.1\times 10^{-6}$	67.57	1.8
10	L70P30(ILE40)(PC5)Li		$4.8\times 10^{-7}$	7.50	1.4
11	L65P35(ILE40)(PC5)Li		$1.2\times 10^{-6}$	30.44	1.2
12	L70P30(ILE40)Li(Al2)	$2.0\times 10^{-5}$	$3.6\times 10^{-7}$	4.61	2.4
13	L70P30(ILE45)Li(Al2)	$2.5\times 10^{-5}$	$4.2\times 10^{-6}$	9.11	2.6
14	L70P30(ILE50)Li(Al2)	$2.7\times 10^{-5}$	$5.7\times 10^{-6}$	2.95	2.3

**Table 4:** Electrochemical and thermomechanical data for the polymer electrolytes.

**Entry**	**Sample**	***T_g_* (** **°C)**	E′ (MPa) (T = 30 °C)	σ_0_ (S·cm^−1^)	σ_1_ (S·cm^−1^)
1 ^1,2^	L65P35(ILE30)	65 ± 2	1202 ± 10	-	$1.3\times 10^{-7}$
2 ^1^	L65P35(ILE40)	68 ± 2	495 ± 60	-	$1.4\times 10^{-5}$
3 ^1^	L65P35(ILE45)	63 ± 3	401 ± 80	-	$4.5\times 10^{-5}$
4 ^1,2^	L65P35(ILE30)Al_2_	83 ± 1	1213 ± 164	$7.0\times 10^{-4}$	$1.6\times 10^{-6}$
5 ^1^	L65P35(ILE40)Al_2_	70 ± 2	461 ± 62	$6.8\times 10^{-5}$	$9.6\times 10^{-6}$
6 ^1^	L65P35(ILE45)Al_2_	65 ± 3	469 ± 89	$1.8\times 10^{-4}$	$1.8\times 10^{-7}$
7	L65P35(ILE30)Li	68 ± 1	1235 ± 20	-	$7.6\times 10^{-8}$
8	L65P35(ILE30)Li(Al_2_)	85 ± 1	1224 ± 103	$1.4\times 10^{-5}$	$5.5\times 10^{-7}$

^1^ No lithium ^2^ Ref. [25].

## Data Availability

The original contributions presented in the study are included in the article/Supplementary Materials, further inquiries can be directed to the corresponding author/s.

## Supplementary Materials

The following supporting information can be downloaded at: https://www.mdpi.com/article/10.3390/polym16142048/s1. Figure S1. Nyquist Plots for electrolytes containing 40 wt % ILE; Figure S2. Nyquist Plots for electrolytes containing 45 wt % ILE; Figure S3. Nyquist Plots for electrolytes containing 50 wt % ILE; Figure S4. Nyquist Plots for electrolytes containing 5 wt %PC; Figure S5. Nyquist Plots for electrolytes containing alumina. (a) Electrolytes 45 wt %ILE and comparison with its analogous without alumina. (b) Electrolytes 50 wt %ILE and comparison with its analogous without alumina. Table S1. Fitting for the equivalent circuit of Nyquist Plot for structural supercapacitor fabricated using L70P30(ILE40)LiAl2 electrolyte.
