# Supplementary material for: Hybrid Solid Polymer Electrolytes Based on Epoxy Resins, Ionic Liquid, and Ceramic Nanoparticles for Structural Applications
Source: Polymers (Basel). 2024 Jul 18;16(14):2048. doi: 10.3390/polym16142048 (PMC11280790; doi:10.3390/polym16142048)
Supplement: Supplementary file 1 [file polymers-16-02048-s001.zip › polymers-3099327-supplementary.pdf]

**Supplementary Materials**

# Hybrid Solid Polymer Electrolytes Based on Epoxy Resins, Ionic Liquid, and Ceramic Nanoparticles for Structural Applications

**Bianca K. Muñoz, Jorge Lozano, María Sánchez and Alejandro Ureña**

**Content**


| | Page |
|---|---|
| **Figure S1.** Nyquist Plots for electrolytes containing 40 wt % ILE........................ | 2 |
| **Figure S2.** Nyquist Plots for electrolytes containing 45 wt % ILE....................... | 2 |
| **Figure S3.** Nyquist Plots for electrolytes containing 50 wt % ILE...................... | 2 |
| **Figure S4.** Nyquist Plots for electrolytes containing 5 wt %PC......................... | 3 |
| **Figure S5.** Nyquist Plots for electrolytes containing alumina. (a) Electrolytes 45 wt %ILE and comparison with its analogous without alumina. (b) Electrolytes 50 wt %ILE and comparison with its analogous without alumina... | 3 |
| **Table S1.** Fitting for the equivalent circuit of Nyquist Plot for structural supercapacitor fabricated using L70P30(ILE40)LiAl2 electrolyte....................... | 4 |

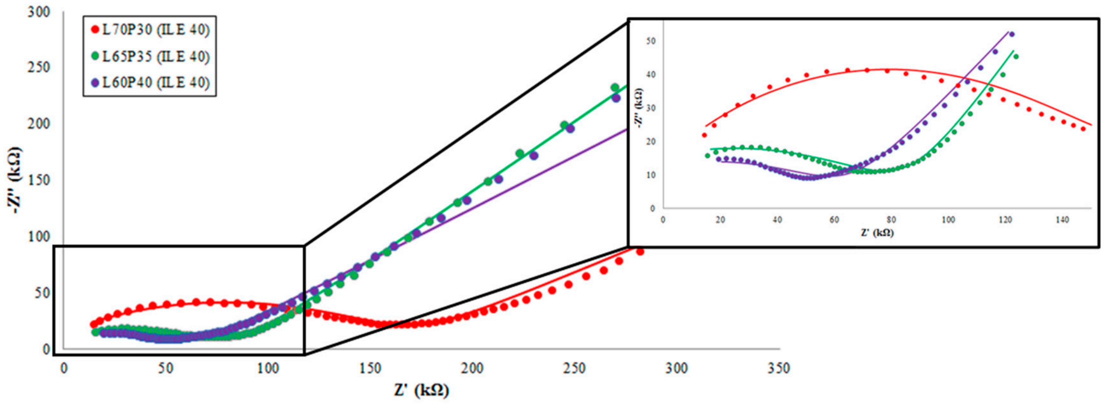


Figure S1. Nyquist Plots for electrolytes containing 40 wt % ILE

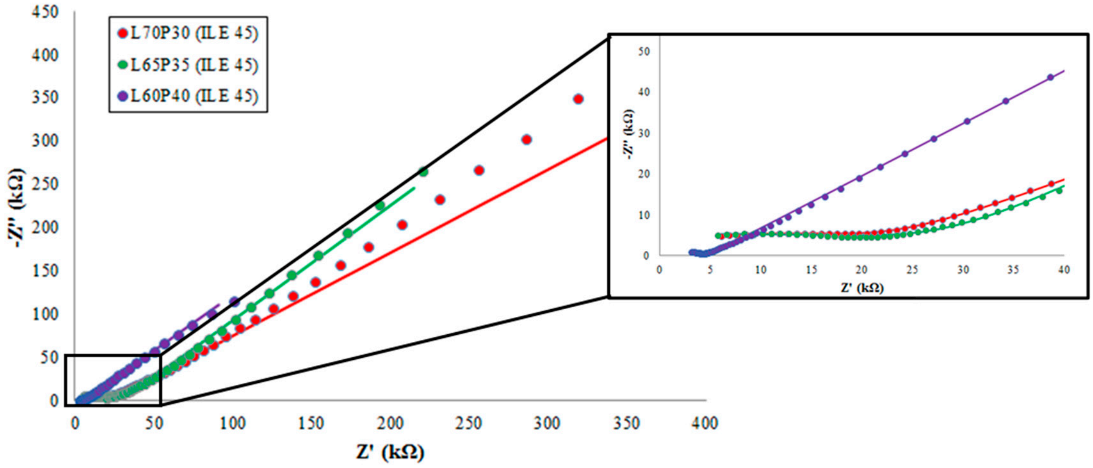


Figure S2. Nyquist Plots for electrolytes containing 45 wt % ILE

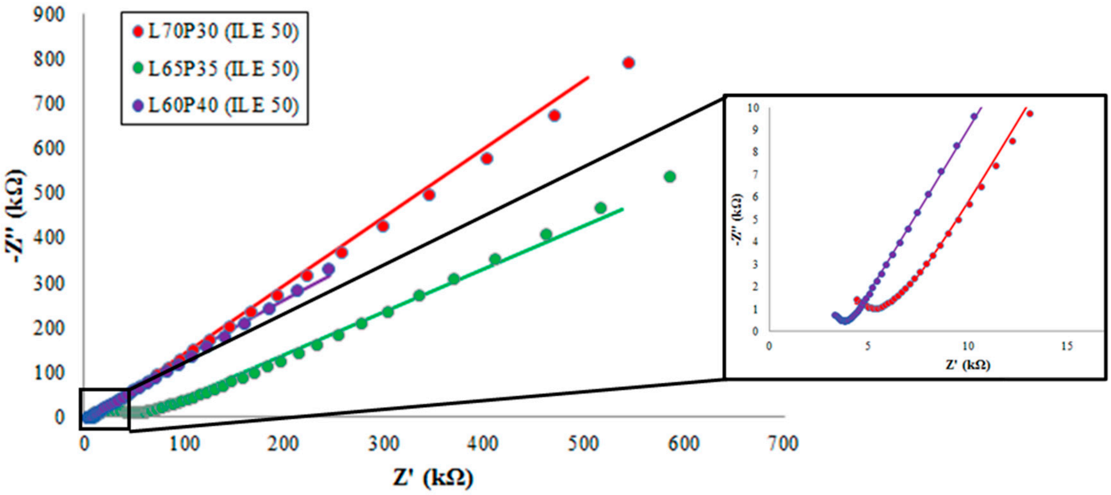


Figure S3. Nyquist Plots for electrolytes containing 50 wt % ILE

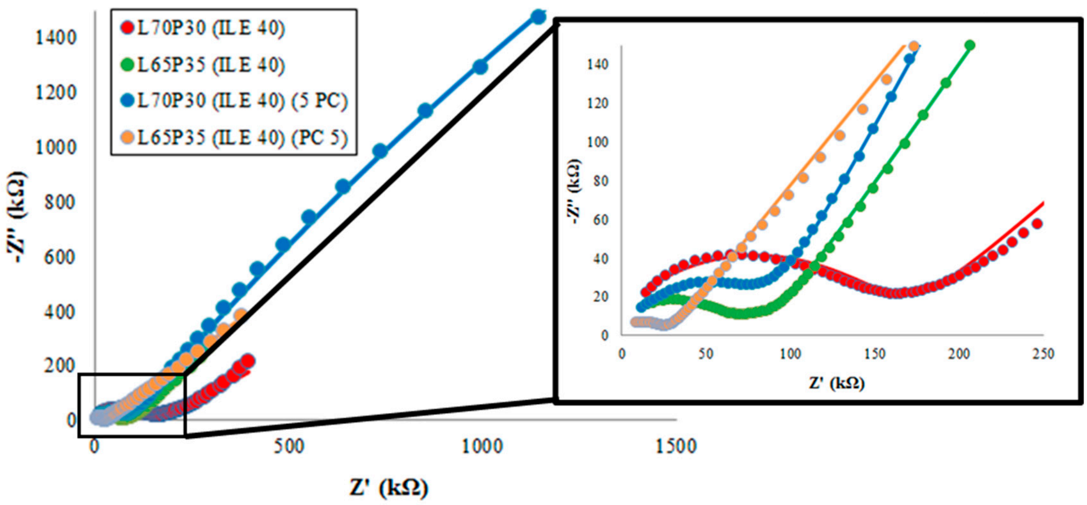


Figure S4. Nyquist Plots for electrolytes containing 5 wt %PC

(a)

(b)

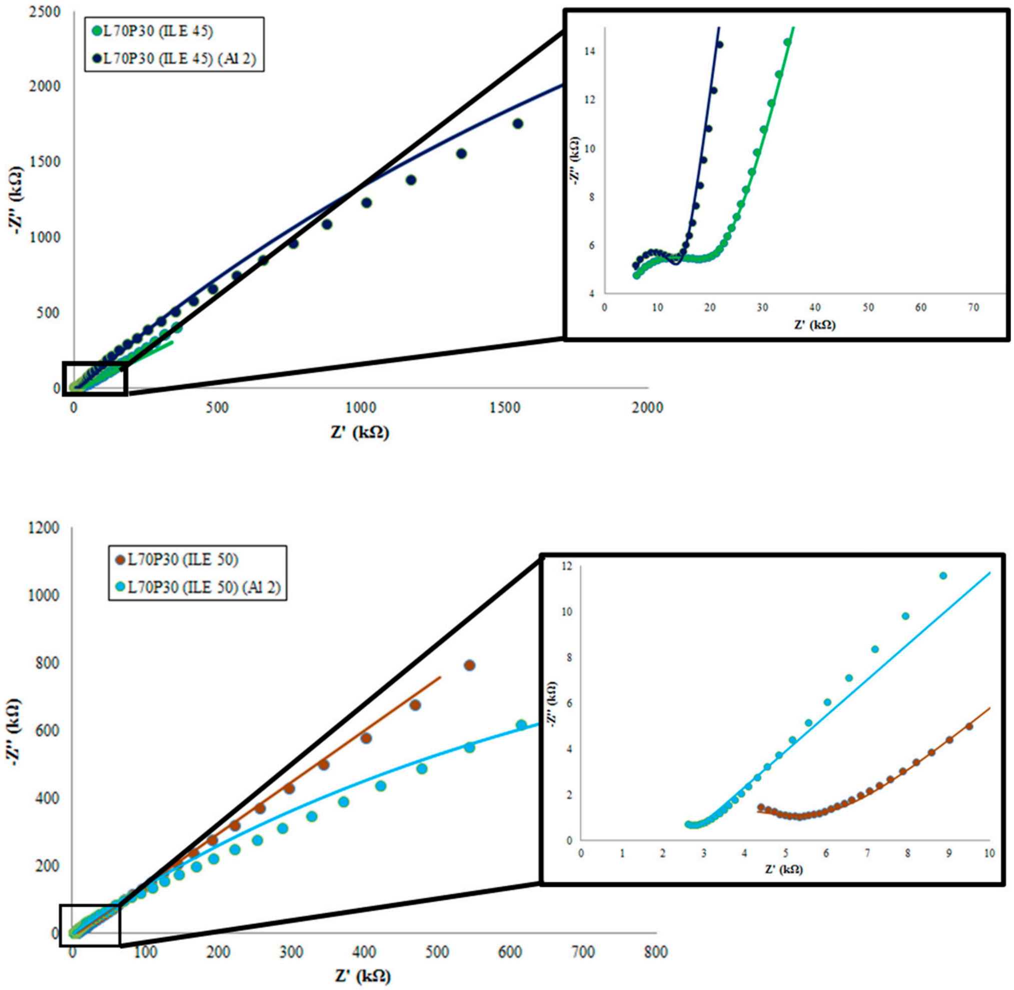


Figure S5. Nyquist Plots for electrolytes containing alumina. (a) Electrolytes 45 wt %ILE and comparison with its analogue without alumina. (b) Electrolytes 50 wt %ILE and comparison with its analogue without alumina.

Table S1. Fitting for the equivalent circuit of Nyquist Plot for structural supercapacitor fabricated using L70P30(ILE40)LiAl2 electrolyte.

| Element | Parameter | Value | Estimated Error (%) |
|---|---|---|---|
| **R1** | **R** | **282,46** | **0,368** |
| **Q1** | **Y0** | **2,2259E-06** | **8,365** |
| | **N** | **0,57906** | **1,139** |
| **W1** | **Y0** | **0,0065913** | **1,833** |
| | $\chi^2$ | 0,018239 | |
